# Detection of doxorubicin, cisplatin and therapeutic antibodies in formalin-fixed paraffin-embedded human cancer cells 

**DOI:** 10.1007/s00418-020-01857-x

**Published:** 2020-03-03

**Authors:** Lukas Böckelmann, Christin Starzonek, Ann-Christin Niehoff, Uwe Karst, Jürgen Thomale, Hartmut Schlüter, Carsten Bokemeyer, Achim Aigner, Udo Schumacher

**Affiliations:** 1 Institute of Anatomy and Experimental Morphology, Center for Experimental Medicine, University Cancer Center Hamburg, University Medical Center Hamburg-Eppendorf, Martinistrasse 52, 20246 Hamburg, Germany; 2 Department of Oncology, Hematology and Bone Marrow Transplantation with Section Pneumology, University Cancer Center Hamburg, University Medical Center Hamburg-Eppendorf, Martinistrasse 52, 20246 Hamburg, Germany; 3 Institute of Inorganic and Analytical Chemistry, University of Münster, Corrensstrasse 28/30, 48149 Münster, Germany; 4 Institute of Cell Biology, Essen University Hospital, Hufelandstrasse 55, 45122 Essen, Germany; 5 Institute of Clinical Chemistry and Laboratory Medicine, Center for Diagnostics, University Medical Center Hamburg-Eppendorf, Martinistrasse 52, 20246 Hamburg, Germany; 6 Rudolf-Boehm-Institute for Pharmacology and Toxicology, Clinical Pharmacology, Härtelstrasse 16-18, 04107 Leipzig, Germany

**Keywords:** Antineoplastic agents, Agar, Cell pellet, DNA adduct, Formaldehyde, Paraffin embedding, Tissue processing

## Abstract

A major limitation in the pharmacological treatment of clinically detectable primary cancers and their metastases is their limited accessibility to anti-cancer drugs (cytostatics, inhibitory antibodies, small-molecule inhibitors) critically impairing therapeutic efficacies. Investigations on the tissue distribution of such drugs are rare and have only been based on fresh frozen material or methanol-fixed cell culture cells so far. In this paper, we expand the detection of cisplatin-induced DNA adducts and anthracyclines as well as therapeutic antibodies to routinely prepared formalin-fixed, paraffin-embedded sections (FFPE). Using pre-treated cell lines prepared as FFPE samples comparable to tissues from routine analysis, we demonstrate that our method allows for the detection of chemotherapeutics (anthracyclines by autofluorescence, cisplatin by immune detection of DNA adducts) as well as therapeutic antibodies. This methodology thus allows for analyzing archival FFPE tissues, as demonstrated here for the detection of cisplatin, doxorubicin and trastuzumab in FFPE sections of tumor xenografts from drug-treated mice. Analyzing human tumor samples, this will lead to new insights into the tissue penetration of drugs.

## Introduction

Many factors exist that affect the response of a tumor to systemically given chemo or antibody therapy. Compared to research on the genetic and cellular level of drug resistance, only little is known about the response to chemotherapy on the tissue level and above. Anti-cancer drugs enter the tumor via the bloodstream and are then distributed within the interstitial compartment. Ideally, this distribution would grant the drug access to the whole tumor mass and thereby all cancer cells. This proposition has been challenged by the finding that monoclonal antibodies can only be detected within an area of 100 µm around the blood vessels, but not beyond (Heine et al. 2011, 2012). This limited access of the antibody was accompanied by significantly increased interstitial fluid pressure (IFP) within the primary tumor. For a growing number of solid cancer entities, elevated levels of IFP have been reported and by hampering convection in the tissue, this might be an important cause of clinical resistance of solid tumors to chemotherapy. Similar observations have been made for the conventional small-molecule anti-cancer drug doxorubicin in tissue samples from human tumor xenograft models (Primeau et al. 2005). Furthermore, chemical interaction of cisplatin with the nuclear DNA can be visualized using adduct-specific monoclonal antibodies (Liedert et al. 2006; Seoane et al. 2019). Both cisplatin and anthracyclines (particularly daunorubicin and doxorubicin) are among the most frequently used chemotherapeutic drugs for a variety of cancer entities and can, therefore, serve as model substances to study drug penetration and distribution in solid tumors (O’Dwyer et al. 2000; Ho et al. 2016). Anthracyclines are cytotoxic mainly due to DNA intercalation and are known to be fluorescent, making them an ideally suited substance class for tissue detection. Cisplatin, on the other hand, exerts its cytotoxic effect via the formation of intra- and interstrand DNA crosslinks. Liedert et al. (2006) described the generation of a highly specific monoclonal antibody, Mab R-C18, detecting guanine–guanine (Pt-[GpG]) intrastrand crosslinks, which constitutes the major cisplatin reaction product. However, all previous approaches to study drug penetration and distribution have used fresh frozen or methanol-fixed material. As most immunohistochemical (IHC) studies on human material are performed using formalin-fixed, paraffin-embedded (FFPE) tissue sections, we wanted to investigate whether the above-mentioned techniques can also be applied to cultured cells processed in this way. We previously developed a technique where culture cells are fixed in formalin, embedded in agar and processed for histology as solid tissue pieces (Schumacher et al. 1994). In this investigation, we show that anthracyclines and cisplatin adducts, as well as therapeutic antibodies, can indeed be detected in FFPE sections of tumor cell lines. We provide data for a broad range of clinically relevant human malignant tumor cell lines, including leukemias as well as adenocarcinomas and squamous cell carcinomas originating from different tissues. In addition, the feasibility of laser ablation–inductively coupled plasma–mass spectrometry (LA–ICP–MS) in detecting cisplatin in FFPE sections is demonstrated. With a high spatial resolution down to low micrometer range, this method enables for quantitatively investigating drug penetration on the tissue level complementing IHC methods.

The herein-described methodology can now be applied to archival FFPE tissues, as demonstrated here for the detection of cisplatin, doxorubicin and the therapeutic antibody trastuzumab in FFPE sections of tumor xenografts from drug-treated mice. Reevaluating FFPE material of xenograft experiments or even of patient material has many advantages. Not only is archival FFPE material readily available in many laboratories, but is also well conserved with paraffin sections providing for higher resolution and superior quality of tissue morphology compared to frozen sections. This can in turn also contribute to the reduction of animal experiments. Furthermore, broad application to clinical samples and biopsies may allow to correlate drug tissue penetration levels to clinical response and outcome.

## Materials and methods

### Drugs and antibodies

Cisplatin (1 mg/ml, Accord Healthcare Limited, North Harrow, United Kingdom), doxorubicin (2 mg/ml, TEVA GmbH, Ulm, Germany), PEGylated liposomal doxorubicin Caelyx^®^ (2 mg/ml, Janssen-Cilag, Beerse, Belgium), trastuzumab (Herceptin^®^, 21 mg/ml, Roche Pharma AG, Grenzach-Wyhlen, Germany), cetuximab (Erbitux^®^, 5 mg/ml, Merck KGaA, Darmstadt, Germany) and rituximab (MabThera^®^, 10 mg/ml, Roche Pharma AG, Grenzach-Wyhlen, Germany) were obtained from the hospital pharmacy as solutions for infusion and stored at 4 °C.

The rat anti-Pt-[GpG] monoclonal antibody R-C18 for the detection of cisplatin DNA adducts was provided by Oncolyze (Essen, Germany) and the biotin-conjugated rabbit anti-rat secondary antibody was purchased from Jackson ImmunoResearch Laboratories (312-065-048, West Grove, PA, USA). Rat IgG2a kappa isotype control was obtained from eBioscience (14-4321-85, San Diego, CA, USA).

As a secondary antibody for the detection of human monoclonal antibodies in tissue, a biotin-conjugated goat anti-human monoclonal antibody (B1140, Sigma-Aldrich, Taufkirchen, Germany) was used. As a control, cells were treated with isotype-matched human IgG1 kappa antibody (731699, Beckman Coulter, Fullerton, CA, USA) instead of the therapeutic antibody.

### Cell lines and cell culture

The human promyelocytic leukemia cell line HL-60, human B-lymphoblastoid cell line IM-9, human Burkitt’s lymphoma cell line Ramos, human triple negative breast cancer cell line MDA-MB-231, human squamous cell carcinoma cell line UTSCC24A and UTSCC2, human small-cell lung cancer cell line OH-1, human prostate cancer cell line PC3, human esophageal adenocarcinoma PT1590, human colorectal adenocarcinoma cell line HT29, human ovarian carcinoma cell lines OVCAR3 and SKOV3 as well as the human melanoma cell line MeWo were analyzed. All cell lines, except for SKOV3, were grown in Roswell Park Memorial Institute (RPMI-1640) medium (Gibco™, Waltham, MA, US). SKOV3 cells were cultured in McCoy’s 5A medium (Gibco™, Waltham, MA, US). For all cell lines, media were supplemented with 10% fetal calf serum (FCS) and 50 units/ml penicillin and 50 µg/ml streptomycin (Gibco™, Waltham, MA, US). The cells grow adherently except for HL-60, IM9, Ramos and OH-1, which were cultured in suspension. All cells were maintained in a humidified atmosphere of 95% air plus 5% CO_2_ at 37 °C.

### Treatment of cells with cytostatic drugs and anti-cancer antibodies

Cells were incubated with 12 µM doxorubicin for 1 h, 12 µM PEGylated liposomal doxorubicin for 12 h or 32 µM cisplatin for 1, 2, 4, 8 and/or 24 h as indicated. Cell lines PT1590, Ramos and UTSCC2 were incubated with 10 µg/ml of monoclonal antibody (trastuzumab, rituximab or cetuximab) or isotype control antibody, respectively, for 30 min. For LA–ICP–MS analysis, UTSCC24A cells were treated with 5, 25 and 40 µg/ml cisplatin for 4 h.

### Fixation and embedding of cells into agar

After treatment with cytostatics, cancer cells were rinsed with phosphate-buffered saline (PBS) (Gibco™, Waltham, MA, US), collected by scraping off the culture flask and pelleted by centrifugation at 365 ×*g* for 10 min. Cells were resuspended in 5 ml of 4% formalin in 0.1 M sodium phosphate buffer and fixed for 20 min at room temperature. Next, the cells were washed twice in PBS using a centrifugation step at 365 ×*g* for 10 min. Until embedding into agar, fixed cells were kept in PBS at 4 °C.

For embedding of cells, 2% Difco™ Noble Agar (Becton, Dickinson, Sparks, MD, USA) was heated up and kept at a temperature of 55 °C afterwards. The cells were sedimented again by centrifugation at maximum speed for 30 s in a tabletop centrifuge and the supernatant was discarded. Cell pellets were resuspended completely in 300 µl of liquid agar in a 1.5 ml reaction tube and the tube was immediately centrifuged at maximum speed for 30 s to form cell pellets again. Afterwards, tubes were cooled on ice. The solid agar piece was carefully removed from the tube and the excess amount of agar was cut off with a scalpel. Agar pellets were then subjected to standardized tissue infiltration using a Leica TP1020 tissue processor (Leica Biosystems, Nussloch, Germany). Subsequent paraffin embedding was performed using a Leica EG1160 Paraffin Embedding Center (Leica Biosystems, Nussloch, Germany).

### Sectioning

Cell pellets were sectioned with a thickness of 4 µm, mounted on HistoBond^®^ glass slides (Paul Marienfeld, Lauda-Königshofen, Germany) and allowed to air-dry, followed by drying in an incubator at 37 °C overnight.

### Cisplatin immunohistochemistry

Formalin-fixed and paraffin-embedded sections were de-paraffinized in two changes of xylene (5 min each) and rehydrated in a series of graded ethanol (100, 96, 70 and 50% for 5 min each). Sections were then washed in aqua dest for 2 min. The following incubation steps were carried out in a moist chamber. For epitope retrieval, samples were treated for 5 min with Fast Enzyme (Zytomed Systems, Bargteheide, Germany) at room temperature, following two 5 min washes in Tris-buffered saline/0.1% Tween20 (TBS-T) and one 5 min wash in TBS (pH 7.6). Blocking with 4% BSA in TBS was performed for 30 min to prevent nonspecific antibody binding. Afterwards, sections were incubated with primary rat anti-Pt-[GpG] monoclonal antibody diluted 1:1000 in antibody diluent (medac, Wedel, Germany) or rat IgG2a kappa at a dilution of 1:500 (eBioscience, San Diego, USA) for 80 min at room temperature and then rinsed twice with TBS-T as well as with TBS for 5 min each. Subsequently, the secondary biotin-conjugated rabbit anti-rat antibody (Dako, Glostrup, Denmark) was incubated at a dilution of 1:100 in antibody diluent for 30 min at room temperature, followed by rinsing twice with TBS-T and once with TBS for 5 min each. Sections were treated with Vectastain^®^ ABC-AP Kit (Vector Laboratories, Burlingame, CA, US) according to the manufacturer’s recommendations for 30 min at RT and again washed in TBS-T and TBS as described above. Finally, alkaline phosphatase enzyme activity was visualized by incubating the sections with Permanent Red solution (Dako, Glostrup Denmark) for 20 min and counterstained with hematoxylin for 4 s, with intermediate washes under running tap water (3 min) and in aqua dest (2 min). Slides were dehydrated in a series of graded ethanol (70% for 15 s, 96 and 100% for 5 min each) and three changes of xylene (5 min each) and finally covered with Eukitt^®^ Mounting Medium (Sigma-Aldrich, Steinheim, Germany) and coverslips.

### Therapeutic monoclonal antibody immunohistochemistry

For FFPE sections, all steps including deparaffinization, rehydration, epitope retrieval with Fast Enzyme, blocking with 4% BSA and washing steps were carried out as described above. Sections were incubated with secondary biotin-conjugated goat anti-human monoclonal antibody (Sigma-Aldrich, Taufkirchen, Germany) diluted 1:200 in antibody diluent for 1 h at room temperature. Subsequently, sections were treated with Vectastain^®^ ABC-AP Kit (Vector Laboratories, Burlingame, CA, US) according to the manufacturer’s recommendations for 30 min at RT and again washed in TBS-T and TBS as described above. Finally, sections were incubated with Permanent Red solution (Dako, Glostrup Denmark) for 20 min. Counterstaining with hematoxylin, dehydration and mounting was carried out as described above. Isotype control antibody-treated cells were subjected to the same procedure.

### Microscopy of immunohistochemical stainings

IHC-processed sections were first evaluated using a ZEISS Axiophot 2 microscope (Carl Zeiss, Jena, Germany). Digital images were obtained with a ZEISS Axio Scan Z1 slide scanner equipped with a ZEISS EC Plan-Neofluar 20×/0.50 Pol M27 objective (Carl Zeiss, Jena, Germany) and a Hitachi HV-F20SCL camera with 1600 × 1200 pixels (Hitachi Kokusai Electric America Ltd., New York, USA). For image acquisition, ZEISS ZEN 2.3 software was used (Carl Zeiss, Jena, Germany). Images were further processed with netScope Viewer software (Net-Base Software, Freiburg, Germany).

### Fluorescence microscopy of doxorubicin

The FFPE sections were de-paraffinized in xylene (two times for 5 min each) and rehydrated in a series of graded ethanols (100, 96, 70 and 50% for 5 min each), ending in aqua dest (2 min). Sections were then mounted with Mowiol^®^ (Sigma-Aldrich, Taufkirchen, Germany), supplemented with 4′,6-diamidino-2-phenylindole (DAPI) (Sigma-Aldrich, Taufkirchen, Germany), for nuclear counterstaining, mounted with a coverslip, and dried overnight in a cooling chamber. Doxorubicin fluorescence was detected using a Nikon Eclipse Ti inverted confocal microscope (Nikon Instruments Inc., Melville, NY, USA). Sections were assessed under 40× magnification and digitally imaged for evaluation of uptake of doxorubicin into the cells. Image analysis was performed using the NIS-elements version 4.20.01 (Nikon Instruments Inc.). DAPI fluorescence representing cell nuclei was visualized with 405 nm excitation and 435–485 nm emission filter sets. Cy3 fluorescence representing doxorubicin was visualized with 561 nm excitation and 572–647 nm emission filter sets. For composite images of doxorubicin and DAPI as well as for measurements of nuclear fluorescence intensity ImageJ 1.48f software was used.

### LA–ICP–MS

Laser ablation–inductively coupled plasma–mass spectrometry imaging was done as previously described for cryostat sections of tumor spheroids (Niehoff et al. 2016). In brief, for quantitative analysis by LA–ICP–MS, a laser ablation system (LSX 213, CETAC Technologies, Omaha, USA) with a 213 nm ns-pulsed Nd:YAG laser equipped with a low-volume custom-built cell was used. All laser ablation parameters were optimized for best signal-to-noise ratio with high spatial resolution. Matrix-matched standards based on gelatin (prepared as previously described) (Niehoff et al. 2016) and tissue sections were ablated line by line (0 µm gap). For the analysis of the respective standards, ten lines were ablated and the mean value as well as the standard deviation was calculated. The ablated material was transported to a quadrupole-based mass spectrometer (iCAP Qc, Thermo Fisher Scientific) working in KED mode with following conditions: PFA MicroFlow nebulizer (Elemental Scientific), cyclonic spray chamber (Thermo Fisher Scientific), quartz injector pipe, Ni sampler and Ni skimmer. An Rh solution (10 ng/l diluted from ICP standard in 2% HNO_3_, SCP Science, Courtaboef, France) was introduced into the ICP–MS simultaneously next to the dry aerosol to monitor the sensitivity of the instrumental setup. The isotope 194Pt was monitored with dwell times adjusted to obtain square pixels for each isotope. Data evaluation was performed using the software ImageJ 1.48f.

## Results

### Doxorubicin fluorescence microscopy

**Fig. 1:**
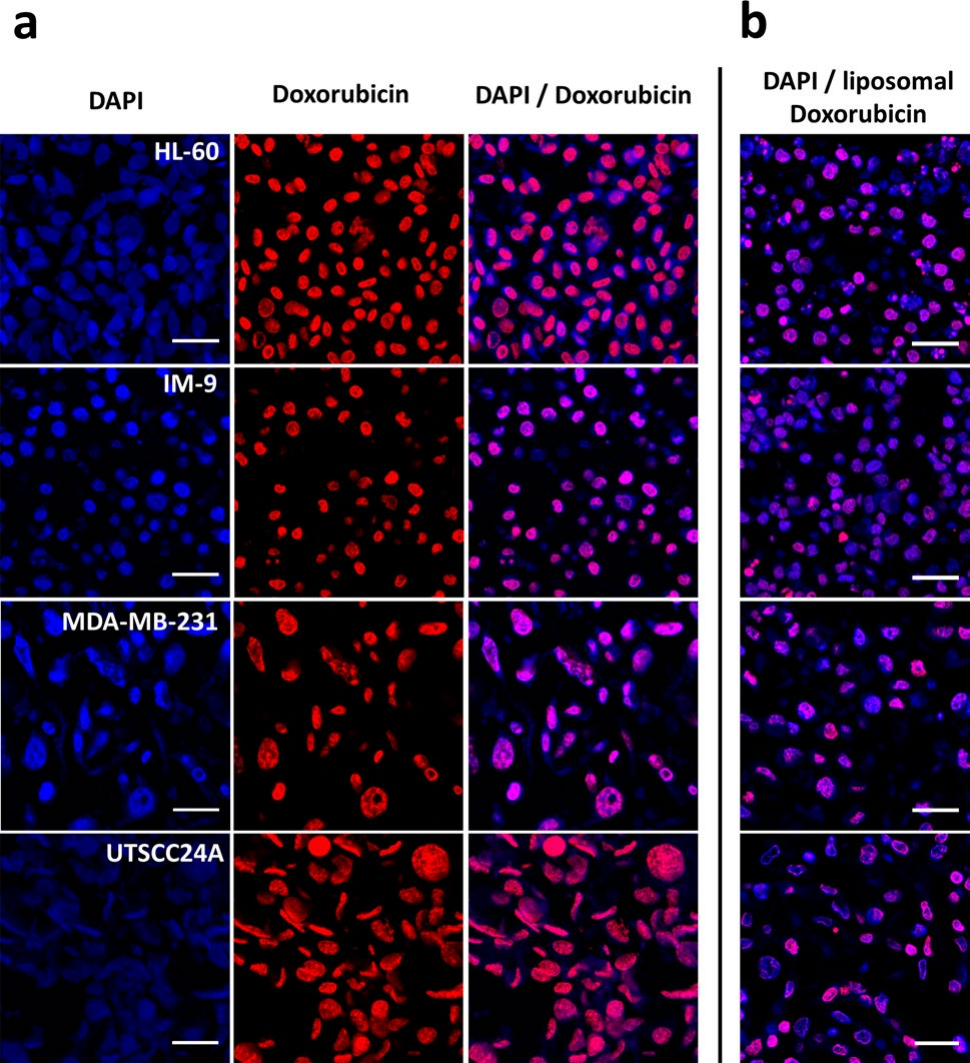
Fluorescence microscopy of doxorubicin, liposomal doxorubicin and DAPI in paraffin sections of different human tumor cell lines. **a** Individual images for DAPI and doxorubicin as well as the merged images are shown for 1 h incubation with 12 µM doxorubicin. **b** For liposomal formulation of doxorubicin, only merged fluorescence images with DAPI staining are shown. Cells were incubated with 12 µM of liposomal doxorubicin for 12 h. *HL-60* acute myeloid leukemia cell line, *IM-9* human B-lymphoblastoid cell line, *MDA-MB-231* human triple negative breast cancer, *UTSCC24A* squamous cell carcinoma of the oral cavity. Scale bar 25 µm

The human promyelocytic leukemia cell line HL-60, human B-lymphoblastoid cell line IM-9, human triple negative breast cancer cell line MDA-MB-231, and human squamous cell carcinoma cell line UTSCC24A were treated with 12 µM doxorubicin for 1 h in culture medium and with 12 µM liposomal encapsulated doxorubicin for 12 h due to its different pharmacokinetics. Subsequently, the cells were subjected to the processing described in the Materials and methods section which resembles the routine standard procedure for tissues, including the subsequent preparation of 4 µm microtome sections. Figure 1a shows the fluorescence microscopy images of doxorubicin autofluorescence and nuclear counterstaining with DAPI as well as the composite images. After 1 h of incubation with doxorubicin, an average of 90% of DAPI-positive cell nuclei were also positive for doxorubicin autofluorescence (HL-60: 90.8%; IM-9: 89.9%; MDA-MB-231: 89.3%; UTSCC24A: 90.8%). However, when treated with liposomal doxorubicin, fluorescence intensity was lower with an average of 72% positive cells (HL-60: 72.5%; IM-9: 76.5%; MDA-MB-231: 60.4%; UTSCC24A: 76.9%). For the liposomal doxorubicin formulation, only composite images are shown (Fig. 1b).

**Fig. 2:**
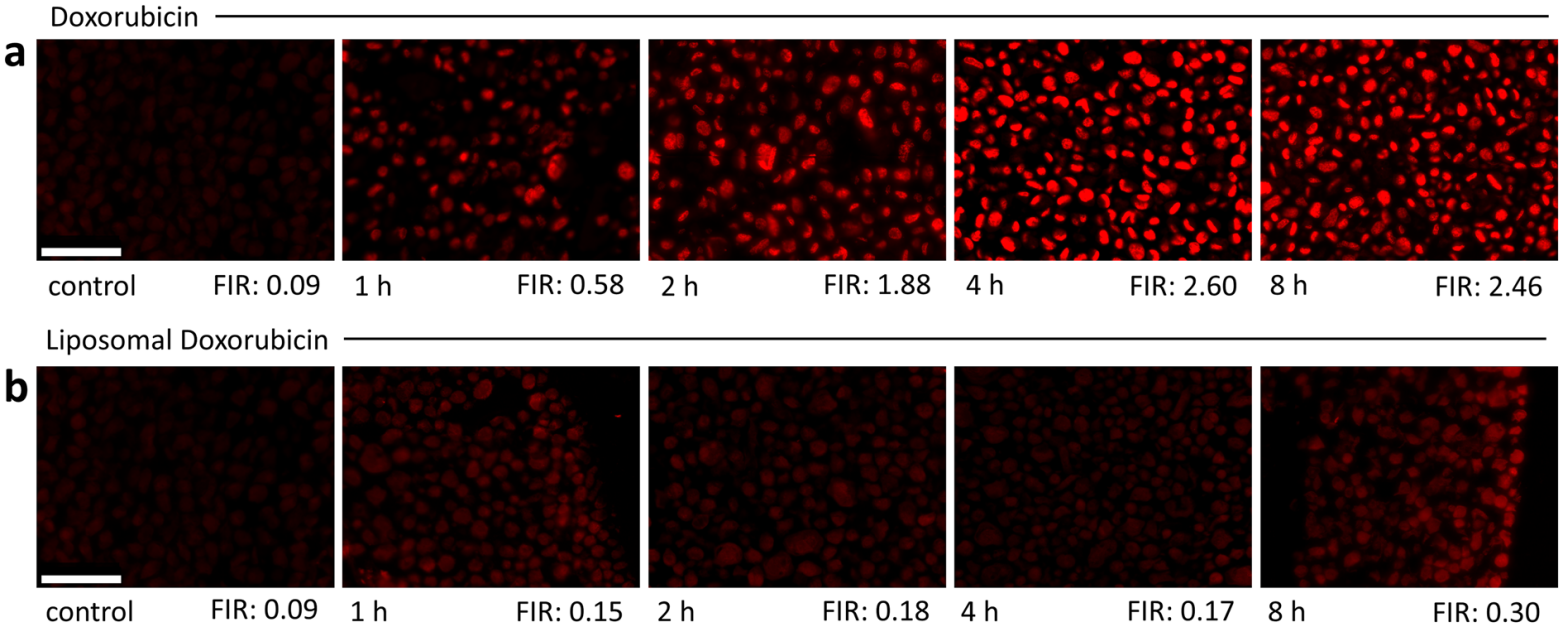
Fluorescence microscopy time series of doxorubicin or liposomal doxorubicin uptake into human tumor cell lines MDA-MB-231. **a** Cells were treated with 12 µM doxorubicin for 1, 2, 4, and 8 h as indicated. **b** Cells were treated with 12 µM liposomal formulated doxorubicin for 1, 2, 4, and 8 h as indicated. Control cells remained untreated. Fluorescence intensity was measured for doxorubicin as well as for DAPI in ten nuclei per time point and mean intensity was calculated. Fluorescence intensity ratio (FIR) was calculated by dividing mean doxorubicin intensity by mean DAPI intensity and is shown for each time point. Scale bar 50 µm

To capture the uptake of doxorubicin or liposomal doxorubicin, respectively, into the nucleus of cells, we carried out a time series experiment (Fig. 2). For doxorubicin, maximal fluorescence intensity was reached after incubation of MDA-MB-231 cells for 4 h with no further increase at the 8 h time point (Fig. 2a). Nuclear uptake of liposomal formulated doxorubicin was somewhat slower with lower fluorescence intensity and showed only a small increase at the 8 h time point (Fig. 2b).

### Detection of cisplatin DNA adduct formation

**Fig. 3:**
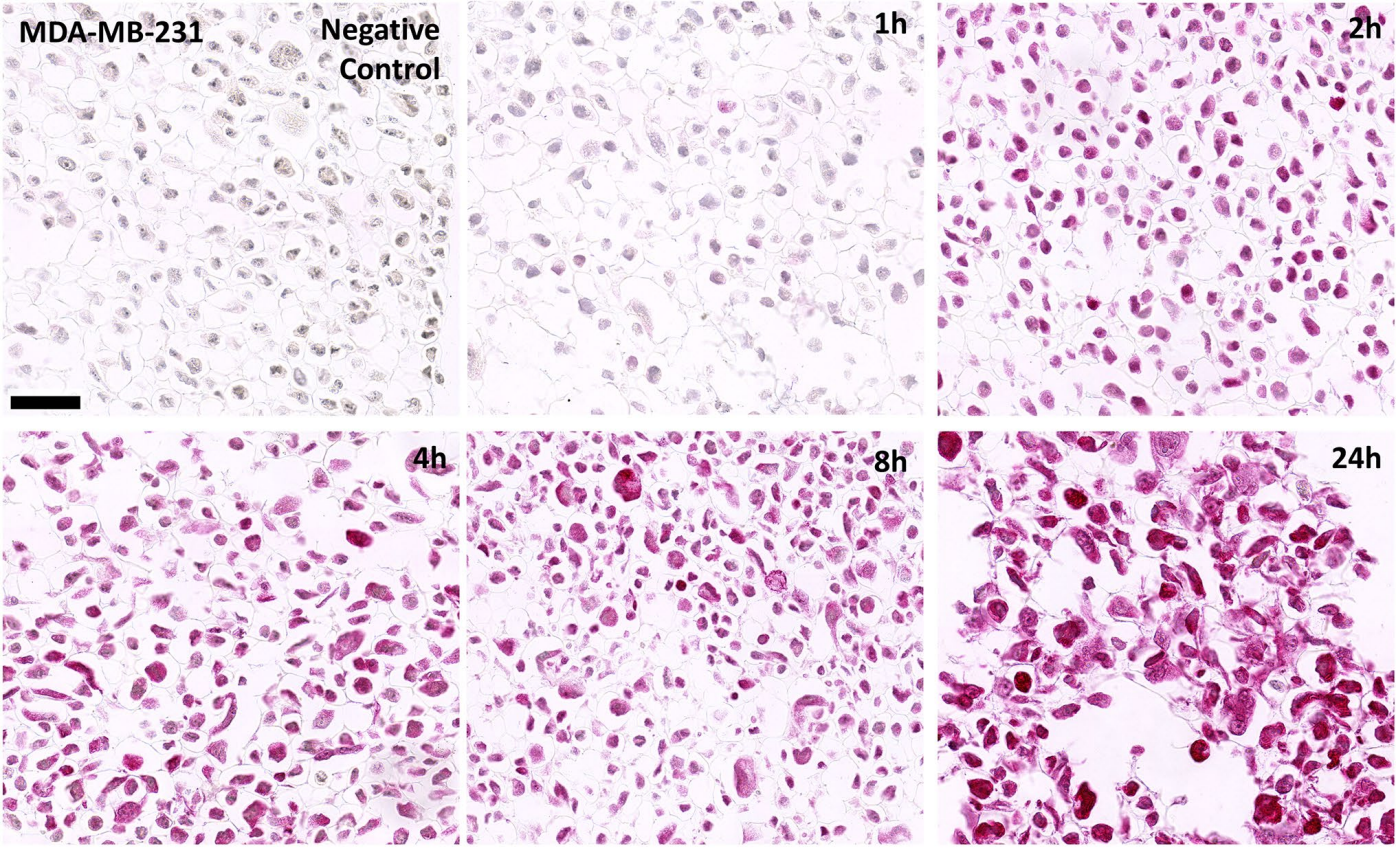
Immunohistochemical detection of cisplatin Pt-[GpG] DNA adducts in paraffin sections of human tumor cell line MDA-MB-231 (human triple negative breast cancer). Cells were incubated with 32 µM cisplatin for 1, 2, 4, 8 and 24 h. Scale bar 50 µm

**Fig. 4:**
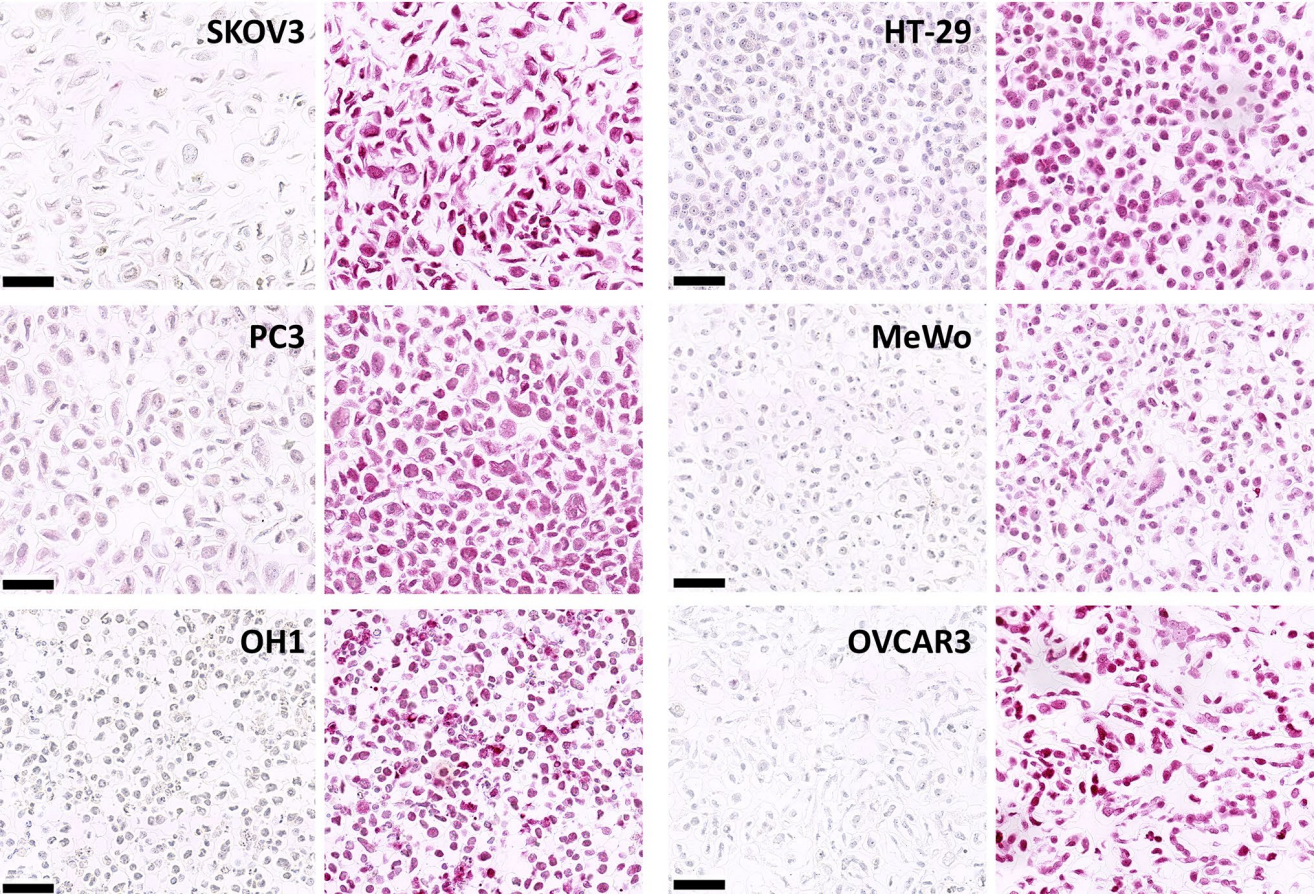
Immunohistochemical detection of cisplatin Pt-[GpG] DNA adducts in paraffin sections of different human tumor cell lines. Cells were incubated with 32 µM cisplatin for 24 h (right images). Untreated cells were used as negative controls (left images). *SKOV3 and OVCAR3* ovarian cancer, *PC3* prostate cancer, *OH1* small cell lung cancer, *MeWo* melanoma, *HT-29* colorectal adenocarcinoma. Scale bar 50 µm

Different strategies of proteolytic epitope retrieval (PIER) for Pt-[GpG] DNA adducts were tested: trypsin, proteinase K and protease cocktail Fast Enzyme. For the treatment with trypsin and proteinase K, only weakly positive signals could be obtained (data not shown). Fast Enzyme yielded strong signal intensity and was, therefore, used in all subsequent experiments as described above. For the human triple negative breast cancer cell line, MDA-MB-231 incubation with 32 µM cisplatin was performed for 1, 2, 4, 8 and 24 h respectively (Fig. 3). After the 1-h incubation with cisplatin, only a minor proportion of tested cells showed a weak staining. A profound increase in the number and signal intensity of positive cells was obtained after 2 h of incubation and thereafter, which is in accordance with formation kinetics of Pt-[GpG] DNA adducts shown in other studies (Nel et al. 2013). Isotype controls were performed with rat IgG2a kappa immunglobuline and showed no detectable signal in IHC. The number of apoptotic cells and cell debris increased over time as well. Increasing apoptosis rates were confirmed by applying IHC for gH2AX (data not shown). The malignant tumor cell lines HT-29, MeWo, OVCAR3, SKOV3, OH1 and PC3 were incubated with 32 µM cisplatin for 24 h. Again, this led to strong signal intensity in all cell lines tested (Fig. 4).

### Validation of cisplatin and doxorubicin detection method in intact tumor tissue

**Fig. 5:**
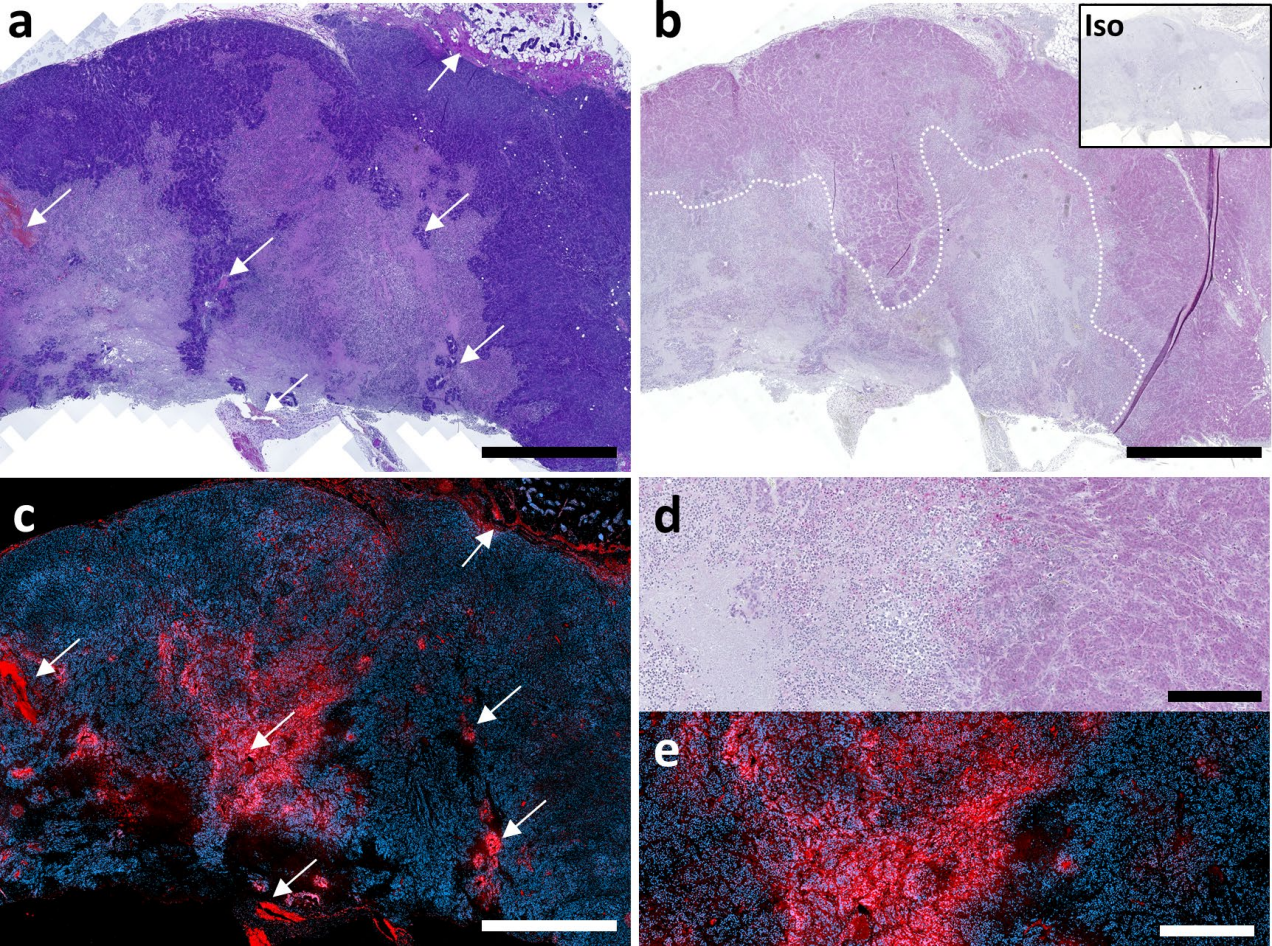
Distribution of cisplatin and doxorubicin in a HT-29 colon adenocarcinoma xenograft tumor. Sections of archival FFPE material of a HT-29 xenograft tumor were subjected either to immunohistochemistry for cisplatin detection or immunofluorescence microscopy to detect doxorubicin. The tumor was subcutaneously grown in an immunodeficient SCID mouse, which was treated with cisplatin and doxorubicin. **a** Microscopic image of H&E stained tumor. Arrowheads point to blood vessels. Scale bar 2 mm. **b** Immunohistochemical detection of cisplatin DNA adduct formation. Isotype antibody control is shown in the upper right corner. Dashed line indicates the border between well-perfused margins of the tumor and the central necrotic region. Scale bar 2 mm. **c** Immunofluorescence image of doxorubicin distribution (red) within the tumor. Nuclei were counterstained with DAPI (blue). Arrowheads point to blood vessels. Scale bar 2 mm. **d** High magnification of marked area in **b**. Scale bar 250 µm. **e** High magnification of marked area in **c**. Scale bar 250 µm

Next, we sought to validate both methods in intact tumor tissue. For this purpose, archival FFPE material was drawn from the repository of the Institute of Anatomy and Experimental Morphology. In a xenograft model, the colon adenocarcinoma cell line HT-29 has been subcutaneously injected into immunodeficient SCID mice. After the tumor had grown, mice have been treated with a chemotherapy regime including cisplatin and doxorubicin. After killing, tumor samples have been formalin-fixed, paraffin-embedded and stored after use. For analysis of drug distribution in tissue sections from these experiments, the same protocols for cisplatin immunohistochemistry and doxorubicin fluorescence microscopy were used as in the cell lines. As depicted in Fig. 5, cisplatin is only distributed in the outer, well-perfused margin of the tumor, while the central and necrotic region remains unreached (Fig. 5b, d). Doxorubicin was detected only in the direct vicinity of blood vessels, while most tumor parts remained unstained (Fig. 5c, e). Vessel were identified in an H&E stained section of the tumor (Fig. 5a).

### Detection of therapeutic antibodies

**Fig. 6:**
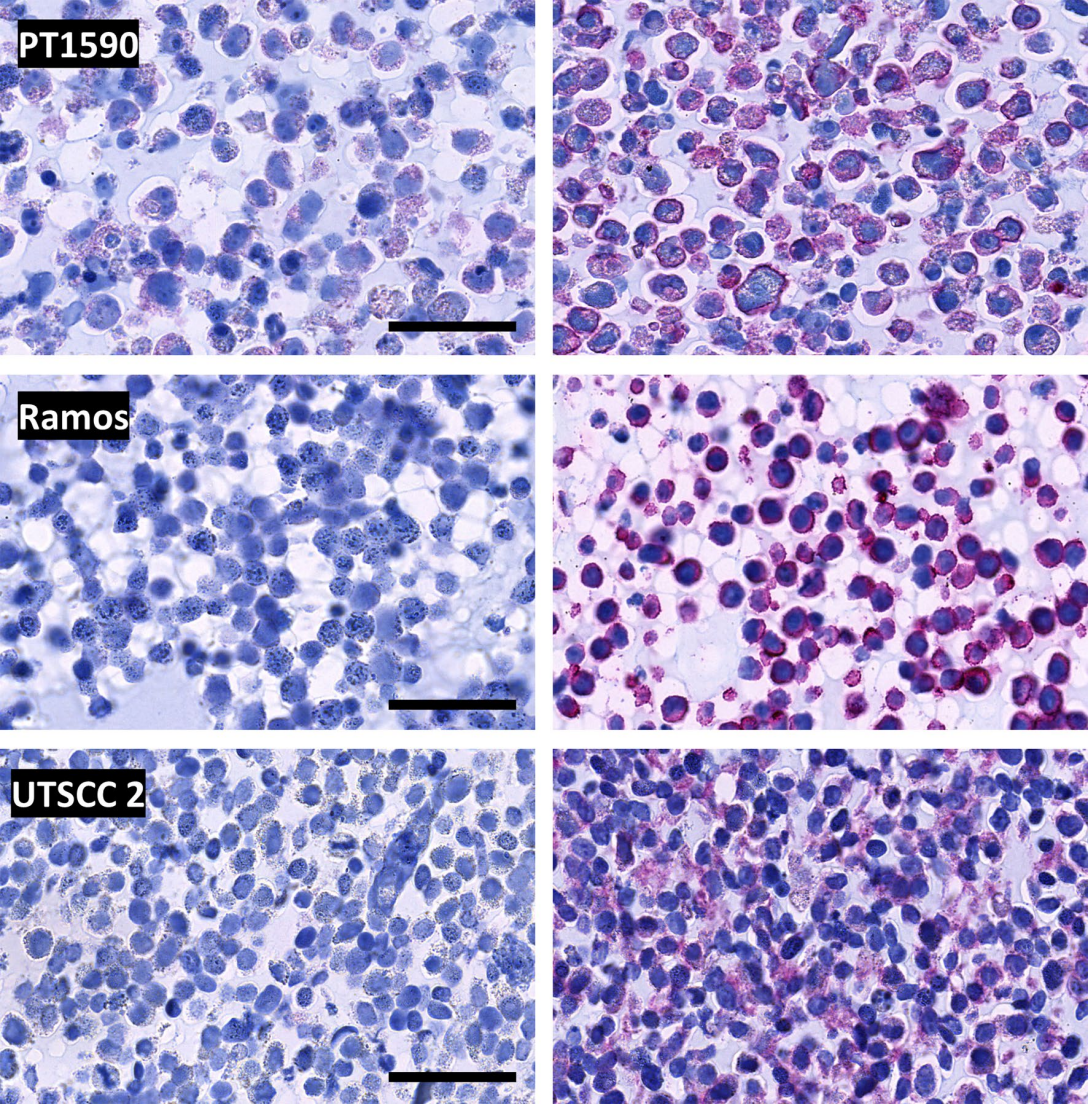
Immunohistochemical detection of therapeutic monoclonal antibodies trastuzumab, rituximab and cetuximab in paraffin sections of human tumor cell lines PT1590, Ramos and UTSCC2, respectively. Microscopic images of isotype-matched human IgG1 kappa antibody treated control cells (left) and cells incubated for 30 min with 10 µg/ml therapeutic monoclonal antibody in cell culture medium (right). *PT1590* esophageal adenocarcinoma, *Ramos* Burkitt’s lymphoma, *UTSCC2* head and neck squamous cell carcinoma. Scale bar 50 µm

We tested for the binding and detection of three frequently used clinically approved monoclonal antibodies in tumor cell lines positive for the respective target: PT1590 cells were treated with trastuzumab targeting the HER2 receptor, Ramos cells with rituximab targeting CD20 and UTSCC2 cells with cetuximab recognizing the EGFR (Fig. 6). After routine fixation of the cells, no non-specific binding or cross-reactivity of the secondary biotin-conjugated goat anti-human monoclonal antibody was observed in control cells. For the PT1590 and Ramos cells, a strong membrane staining for HER2 or CD20, respectively, was observed. In UTSCC2 cells, IHC showed a granular cytoplasmatic staining pattern for the EGFR, indicating an internalization of the receptor-antibody complex upon binding.

### Validation of antibody detection method in intact tumor tissue

**Fig. 7:**
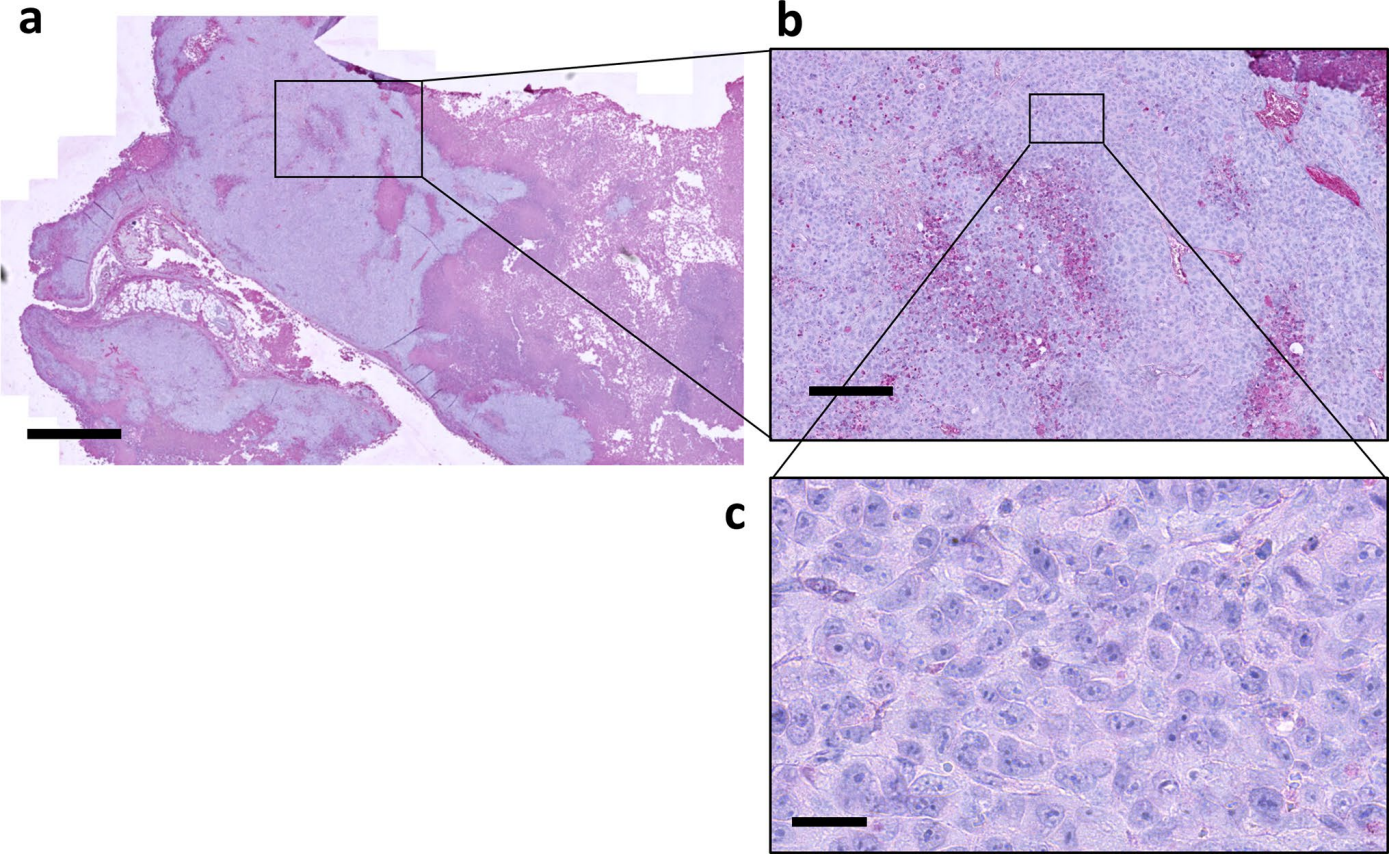
Detection of therapeutic monoclonal antibody trastuzumab in xenograft tumor tissue. **a** Human PT 1590 adenocarcinoma of the gastroesophageal junction xenograft treated several times with anti-human HER2/neu humanized mouse monoclonal antibody trastuzumab (Herceptin^®^). The formalin-fixed, paraffin-embedded tumors were stained for human immunoglobulins. Note that the immunoreactivity is limited to the areas of the blood vessels, particularly in the region of necrotic areas and the connective tissues. Most of the tumor cells remained unstained. Low-power magnification. Scale bar 1000 µm. **b** High-power magnification of tumor cells bordering the necrotic area with absence of anti-human immunoglobulin immunoreactivity. Scale bar 200 µm. **c** Within the central area of the tumor mass, tumor cells are not reached by therapeutic antibody trastuzumab. Scale bar 20 µm

The developed method was then applied to archival tumor material: FFPE sections of human PT 1590 adenocarcinoma of the gastroesophageal junction xenografts were obtained from previously published experiments (Lange et al. 2011). Mice had been treated several times with anti-human HER-2/neu humanized mouse monoclonal antibody trastuzumab (Herceptin^®^). As demonstrated in Fig. 7, the immunoreactivity for human immunoglobulins was limited to the vicinity of the blood vessels, particularly in the region of necrotic areas and the connective tissue capsule of the tumor. Most of the tumor cells remained unstained.

### LA–ICP–MS

**Fig. 8:**
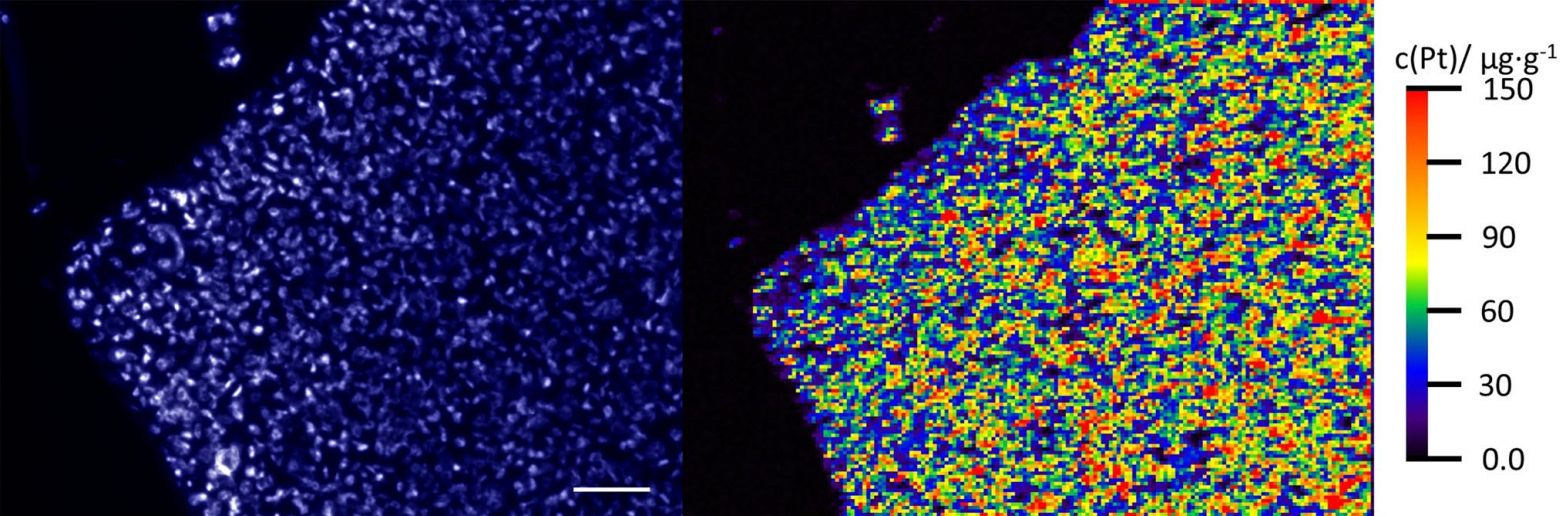
Laser ablation–inductively coupled plasma–mass spectrometry imaging of human tumor cell line UTSCC24A (squamous cell carcinoma of the oral cavity). Fluorescence microscopic image of DAPI nuclear staining (left) and distribution of cisplatin after incubation with 40 µg/ml (133.3 µM) for 4 h. LA–ICP–MS images were obtained with a spatial resolution of 5 µm. Scale bar 100 µm

In a previously published study, Niehoff et al. (2016) demonstrated the feasibility of LA–ICP–MS quantitatively bioimaging for the detection and quantification of platinum group elements (Pt) in cryostat sections of drug-treated tumor spheroids. The same quantification strategy using gelatin-based matrix-matched standards was adopted for the analysis of Pt in FFPE sections of cisplatin-treated tongue squamous cancer cell line UTSCC24A. The method was applied with high spatial resolution of 5 µm. Cells were incubated for 4 h with 40 µg/ml (133.3 µM) cisplatin and routinely processed for formalin fixation and paraffin embedding. As depicted in Fig. 8, platinum concentrations of up to 150 µg/g tissue were ultimately measured in FFPE sections. We also tested for incubation with 25 µg/ml (83.3 µM) and 5 µg/ml (16.7 µM) cisplatin for 4 h with correspondingly lower platinum uptake into the cells (up to 100 µg/g tissue and up to 10 µg/g tissue respectively, data not shown).

## Discussion

In this contribution, new methods for the histochemical localization of cisplatin, the anthracycline doxorubicin and therapeutic monoclonal antibodies in routinely prepared FFPE sections of drug-treated human tumor cell lines are described. These methods were validated in archival primary tumor tissue of drug-treated tumor-bearing mice. In the tumor tissue tested, a clear distribution deficit was observed sparing the central regions of the tumor distant from blood vessels. This held true not only for the macromolecular monoclonal antibody trastuzumab (see Fig. 5), but also for the small molecular substances, cisplatin and doxorubicin (see Fig. 7).

Methods published until now have used either freshly prepared and methanol-fixed cells or immediately prepared cryostat sections of tumor material (Primeau et al. 2005; Heine et al. 2011, 2012; Seoane et al. 2019). The herein described methodology allows for studying FFPE archival material and thus to reevaluate previous experiments. A major advantage of using paraffin sections over frozen ones is that paraffin sections can be routinely cut a 2–4 µm, while frozen sections are generally at least 10 µm thick. Both, the higher resolution and the superior quality of the morphology in paraffin sections are of particular advantage in searching for disseminated tumor cells or small metastatic deposits in the lungs, bone marrow or other metastatic sites. Since FFPE tissue is the most common method of tissue preparation for diagnostic histopathology and is performed in today’s laboratories almost completely automated, the herein introduced protocol enables for large-scale study of drug uptake into cancer cells. In particular, serial sections can be stained for the expression of proteins associated with drug response or drug resistance or even double-staining procedures can be applied to these sections.

It is perhaps not surprising that proteins such as the therapeutic monoclonal antibodies used in our studies can be detected immunohistochemically after conventional antigen retrieval techniques, however, small molecules such as cisplatin and the anthracyclines are normally washed out during fixation and embedding. As anthracyclines are modestly lipophilic (Gallois et al. 1998), they may be similarly preserved by formalin fixation as in the case of amphiphilic gangliosides (Schwarz and Futerman 1997). However, the main effect of their preservation in FFPE tissues is presumably due to the fact that both cisplatin and anthracyclines are so stably incorporated into the DNA that this integration survives paraffin wax processing.

Correlating the actual drug distribution data with other surrogate markers of drug penetration and its cytotoxic efficacy such as apoptotic cells or cell cycle markers will further elucidate the role of drug resistance on the tissue level. One possible application thus can be the examination of tumor samples resected after heated intraoperative chemotherapy (HIPEC) of patients suffering from peritoneal carcinomatosis. Bianga et al. (2014) investigated the distribution of cisplatin in such samples using MALDI and LA–ICP techniques, but in only a small cohort of patients and using fresh frozen samples only. The use of archival FFPE material would allow for the analysis of a greater number of patient samples and the possibility to further correlate drug distribution with survival rates. The applicability of such correlation studies was shown by Kim et al. (2012). They measured tissue platinum concentration in fresh frozen advanced non-small-cell lung cancer (NSCLC) samples from patients who underwent neoadjuvant chemotherapy using flameless atomic absorption spectrophotometry (FAAS). Tissue platinum concentration was significantly associated with tumor response and survival in those patients and reduced drug accumulation may serves as a resistance mechanism in clinical tumor specimens. Access and analysis of archival FFPE samples of such patients would greatly help to foster the clinical significance of drug distribution as an independent marker for treatment efficacy and clinical outcome.
